# Multiscale Patterns of Bacterial and Protist Diversity Across Red Sea Coral Reefs

**DOI:** 10.3390/microorganisms13112549

**Published:** 2025-11-07

**Authors:** Christopher A. Hempel, Larissa Frühe

**Affiliations:** Marine Science Program, Biological and Environmental Science and Engineering Division (BESE), King Abdullah University of Science and Technology (KAUST), Thuwal 23955-6900, Saudi Arabia

**Keywords:** environmental DNA, coral reef microbes, marine protists, marine bacteria, supervised machine learning, indicator species, coral reef monitoring

## Abstract

Coral reef microbial communities play pivotal roles in ecosystem functioning but remain understudied, particularly across spatial gradients and domains. Here, we use environmental DNA (eDNA) metabarcoding of 16S and 18S rRNA genes to profile bacterial and protistan communities in surface sediments from six coral reefs along the central Red Sea. At each reef, we sampled both exposed (seaward) and sheltered (shoreward) sites, enabling a multiscale analysis of diversity and community composition. We found significant differences in alpha and beta diversity between reefs and between exposure sites within reefs for both microbial groups. Redundancy analysis (RDA) and PERMANOVA revealed reef identity and exposure category as key structuring factors. Indicator species analysis and Random Forest classification identified microbial taxa predictive of exposure gradients, with several exact sequence variants (ESVs) serving as robust bioindicators in both methods. Bacterial and protistan communities exhibited overlapping but distinct patterns, highlighting their complementary ecological roles. Our results underscore the importance of fine-scale habitat heterogeneity in shaping reef microbial assemblages and support the integration of multi-domain eDNA data into coral reef monitoring frameworks.

## 1. Introduction

Coral reefs are among the most biologically diverse and ecologically and economically valuable ecosystems on Earth [1,2]. While the visible biodiversity of corals and reef fishes has been extensively studied, these habitats also support vast and complex communities of microorganisms, including bacteria and protists, which play fundamental roles in reef productivity and function [3]. Microbial communities contribute to primary production, nutrient cycling, host symbioses, and food web dynamics, ultimately shaping the health and resilience of reef ecosystems. Despite their importance, reef-associated microbial communities—particularly microeukaryotic protists—remain underrepresented in biodiversity and monitoring studies [4].

Many studies investigating the role of microbes in coral reefs focus on the coral microbiome [5,6,7,8,9] or bacterioplankton assemblages [10]. Yet the highly diverse benthic bacterial communities in reef systems are especially key-players in the benthic–pelagic link [11] and provide a close-knitted interaction between the coral themselves by degrading sinking coral mucus and contributing to the small-scale nitrogen cycle of coral reefs [12,13].

Tropical reef ecosystems sustain a diverse and dynamic microbial community, both associated with corals and the water column [14]. The microbial communities and processes of coral reefs have been investigated in depth, along various reef types, impact statuses, and oceans, for more than 50 years [15,16,17,18]. Especially in shallow reefs, the entirety of microbial communities consists of three sub-communities, benthic bacteria, the bacterioplankton of the water column, and the coral-associated microbiome, each influencing each other [19,20].

Bacterial communities in reefs have been increasingly profiled using high-throughput sequencing, revealing patterns related to reef zone, host association, and environmental conditions [3,21,22]. Protists, by contrast, are often omitted or pooled under broad microbial categories, though they include diverse photoautotrophs, grazers, and parasites [4]. Studies that simultaneously examine both bacteria and protists are rare, yet such comparisons are essential for understanding community assembly processes across microbial domains. Furthermore, few investigations have assessed microbial diversity at multiple spatial scales, leaving open questions about how microbial communities vary not just between reefs but within them.

The shallowest and most thermally tolerant [23] reef systems globally are located in the Red Sea, a semi-enclosed basin characterized by high salinities [24] and elevated temperatures [25], within steep environmental gradients [26,27,28] across the whole Red Sea. However, microbial studies in the Red Sea remain sparse, and even fewer focus on free-living benthic microbial communities independent of host organisms. There is a particular need to understand how microbial communities respond to spatial variation in reef structure and exposure, especially given growing interest in microbial indicators of reef condition.

Here, we use environmental DNA (eDNA) metabarcoding to investigate bacterial and protist community structures across six coral reefs in the central Red Sea. At each reef, we sampled exposed (seaward) as well as sheltered (shoreward) sites, allowing us to test for microbial differentiation at two nested spatial scales: between reefs as well as between exposure categories within reefs. Sheltered and exposed reef sites of reef structures often differ in hydrodynamic forcing, temperature ranges, and nutrient availability [29,30], offering two distinct niches within the same ecosystem. We calculated diversity metrics, performed community composition analyses, and utilized both indicator value analysis and Random Forest classification models to identify key taxa associated with specific reefs and exposure sites (sheltered vs. exposed). These methods are increasingly applied in the monitoring of anthropogenic impacts [31,32,33] but remain underutilized in reef microbial ecology and for the monitoring of reef structures and health. These analyses provide insights into reef microbial biodiversity and aid in the understanding and ultimately protection of these unique communities.

## 2. Materials and Methods

### 2.1. Sampling Stations

**Figure 1:**
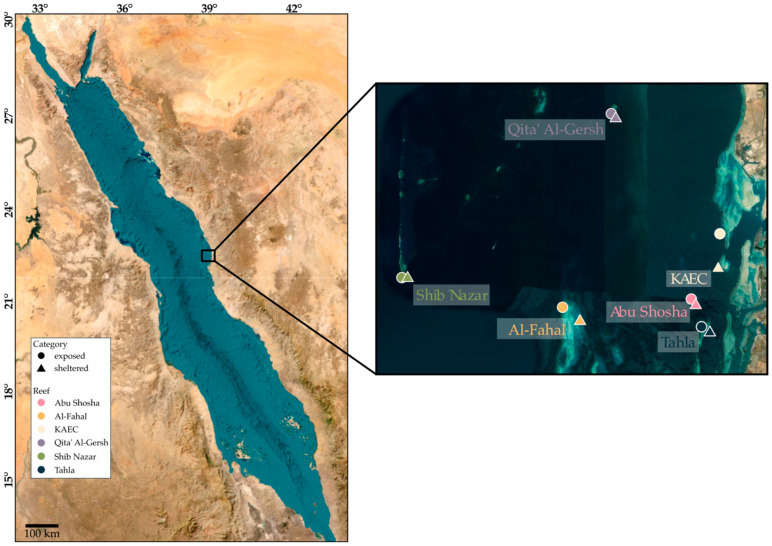
Overview of coral reef sampling locations at the Saudi Arabian Eastern Red Sea coast. Individual dots highlight the locations of the six reefs, and six stations were sampled in triplicate within each reef (three exposed sites and three sheltered sites), totaling 108 individual samples. The average sampling depth was 9 m at Abu Shosha, 7 m at Al-Fahal, 8.5 m at KAEC, 24.5 m at Qita’ Al-Gersh, 21 m at Shib Nazar, and 5.5 m at Tahla. The sampling depth varied between exposed and sheltered sites; for more details, see the Supplementary Table S1.

We sampled six coral reefs (Tahla, Al-Fahal, Shib Nazar, Qita’ Al-Gersh, Abu Shosha, and King Abdullah Economic City (KAEC)) in the central Red Sea along the Saudi Arabian coast (Figure 1). At each reef, six stations were sampled in total: in detail, three at sheltered (shoreward) and exposed (seaward) sites, respectively. Metadata including GPS location and observed environmental parameters are listed in Supplementary Table S1. At each station, surface sediment (max. 5 cm depth) was sampled by scuba divers using 50 mL sterile prelabelled VWR^®^ polypropylene centrifuge tubes. The tubes remained closed underwater until sampling and were closed immediately after sediment was sampled by pushing the sterile tube approx. 5 cm into the sediment and scooping upwards. Divers avoided touching the inside of the tubes during the sampling process. Afterwards, tubes were placed in individual labeled bags and kept in coolers for transport (approx. 2 h). Samples were further processed in a molecular laboratory at King Abdullah University of Science and Technology (KAUST), and seawater was first discarded, followed by the addition of 4 mL of LifeGuard solution (Qiagen, Hilden, Germany) and storage at 4 °C until further processing.

### 2.2. Laboratory Processing

Environmental DNA was extracted from each sample using the PowerSoil Extraction Kit (Qiagen, Hilden, Germany) following a modified version of the manufacturer’s protocol, in which initial bead beating lysis was increased to 20 min. The quality and quantity of extracted eDNA were measured using a Qubit fluorometer (Invitrogen, Waltham, MA, USA) and Nanodrop spectrophotometer (Thermo Fisher Scientific, Waltham, MA, USA). Subsequently, eDNA was amplified using PCR (polymerase chain reaction) with two sets of primers that targeted different barcodes (16S27F534R and 18SV9M, Table 1). Amplification and sequencing were carried out by eDNAtec at the Centre for Environmental Genomics Applications (CEGA), St. Johns, NL, Canada. The datasets were amplified separately and then paired-end sequenced on an Illumina NovaSeq6000 platform with 2 × 150 bp (18SV9M) and 2 × 250 bp (16S27F534R) read lengths.

### 2.3. Bioinformatics Processing

Demultiplexing, adaptor trimming, and primer trimming were carried out by the sequencing facility. We processed both datasets into ESVs with the metabarcoding pipeline Apscale v1.6.3 [37], which involves vsearch v2.20.0 [38], cutadapt v4.4 [39], and a python adaptation of LULU [40]. We ran the Apscale pipeline with a mean expected error cutoff value of 2 (as opposed to the default value of 1, which keeps more erroneous reads during the quality filtering step) and otherwise default parameters.

Taxonomic assignments of ESVs were obtained using BLAST v2.15.0 [41] against the SILVA v138.1 SSU Ref NR99 reference database [42] for bacteria and the PR2 v5.0.0 SSU reference database [43] for protist sequences with an E-value threshold of 1 × 10^−5^. We filtered BLAST v2.15.0 hits as follows: (1) all hits with a bitscore <150 and an alignment length of <100 were excluded; (2) for every ESV, all hits whose bitscore did not fall within a 2% margin of the highest bitscore were excluded; (3) if multiple taxa occurred among the remaining hits, only their Lowest Common Ancestor was retained; and (4) taxonomic lineages were trimmed based on percentage identity to the query; specifically, we trimmed at 100%, 95%, 90%, 85%, 80%, and 75% for the species, genus, family, order, class, and phylum levels, respectively, meaning that only hits with a percentage identity score of >95% were assigned to the genus level and so forth. This stringent filtering approach ensured the minimization of false-positive detections.

### 2.4. Data Analysis

All data analysis was performed in R_4.3.1(2023-06-16)–“Beagle Scouts” using RStudio_2023.06.1+524. All code and supporting files are publicly available and can be found on GitHub (https://github.com/lexscience/Molecular-Coral-Reef-Inventories). Sequence data was deposited in the Sequence Read Archive (SRA) of NCBI under project accession number PRJNA1000118.

The produced ESV-to-sample matrices were stored in a phyloseq object using the phyloseq package v1.44.0 [44]. Using the decontam package v1.20.0 [45], potential contaminant sequences from blanks and negative controls were removed from the datasets. Further, taxonomic hits were checked for each dataset and only sequences with target taxonomy were kept for further analysis (e.g., phylum level hit = “Bacteria” for the bacterial dataset). Exact taxonomic filtering parameters are listed in the code available on GitHub. All sequences assigned to *Homo sapiens* were considered contamination and were removed from all datasets.

#### 2.4.1. Taxonomic Overview Visualization

To visualize microbial community composition across reefs and categories, we generated stacked barplots of relative taxonomic abundance at the phylum level using phyloseq. ESVs were agglomerated at the phylum rank using the tax_glom() function. The data were then transformed to relative abundances using transform_sample_counts() by dividing each taxon’s abundance by the total reads per sample. The resulting community profiles were plotted with ggplot2 v3.5.1., grouped by both reef and category. Taxa with very low relative abundance across samples (<1%) were grouped into the category “Other” to improve visual interpretability.

#### 2.4.2. Alpha Diversity Statistics

Alpha diversity measures (observed richness, Shannon diversity, and Simpson diversity) were calculated for bacterial and protist communities using the estimate_richness() function. Boxplots were generated to visualize alpha diversity distributions across reefs and exposure sites. Kruskal–Wallis tests (non-parametric ANOVA-equivalent) were used to test whether alpha diversity metrics differed significantly among reefs within each exposure site. This was performed separately for each metric and category. Following significant Kruskal–Wallis results, pairwise Wilcoxon rank-sum tests with Benjamini–Hochberg *p*-value adjustments were performed to identify which reefs differed significantly in alpha diversity within each reef exposure site.

#### 2.4.3. Beta Diversity Statistics

To assess differences in microbial community composition, we conducted permutational multivariate analysis of variance (PERMANOVA) using the vegan v2.6-4 [46] and phyloseq packages. We first tested for within-reef differences by grouping samples by reef and running PERMANOVA for each reef separately to evaluate the impact of exposure category on community composition. Next, we performed a between-reef PERMANOVA across all samples to assess differences among reefs irrespective of exposure category. Additionally, to test for combined and interactive effects of reef identity and exposure category (sheltered vs. exposed), we conducted a two-way PERMANOVA using CLR-transformed community data and Euclidean distances. Homogeneity of dispersion was evaluated for each factor using betadisper and permutest, confirming that multivariate dispersion did not differ significantly among groups. All analyses used 999 permutations for significance testing.

#### 2.4.4. Redundancy Analysis

We performed redundancy analysis (RDA) to identify and visualize environmental variables (depth) and categorical variables (exposure category and reef) that significantly impacted community composition. Therefore, ESV abundances were grouped at the phylum level, low-abundance ESVs were removed, and abundances were center log-ratio (CLR) transformed to account for the compositional nature of sequencing data and to enable Euclidean-based ordinations following recommendations by Gloor et al. [47]. Significant environmental variables were identified by performing stepwise selection starting from a null RDA model (with no constraints), using the ordistep() function in both directions with 999 permutations. A full RDA model with all environmental variables as constraints defined the scope of selectable environmental variables. The RDA was then plotted with only the significant variables as constraints.

#### 2.4.5. Indicator ESV Analysis

We used two methods to identify ESVs that significantly indicated either exposed or sheltered reef sites, specifically the indicator value approach [48] and Random Forest (RF) classification [49]. For the indicator value approach, we used the multipatt() function from the indicspecies R package v1.7.15 [48] based on the IndVal.g method with 999 permutations. Taxa with statistically significant associations (*p* < 0.05) were retained. The results were visualized by plotting the top 20 indicator ESVs with the highest indicator values (stat) for bacteria and protists separately, annotated with their full taxonomic classification (phylum and species level), and highlighted by the associated exposure category (sheltered or exposed). For RF classification, ESV tables were extracted from both bacterial and protistan phyloseq objects, and ESVs with zero counts across all samples were removed. RF classification was performed using the randomForest v4.7-1.2 R package [50] with 500 trees and otherwise default parameters. The RF model was trained using exposure category (sheltered or exposed) as the response variable and ESV abundance as the predictor variable. Model performance was assessed using the out-of-bag (OOB) error rate. Variable importance was determined based on the Mean Decrease in Accuracy (MDA). To quantify misclassifications, confusion matrices were generated for both bacterial and protistan datasets.

## 3. Results

### 3.1. Community Composition Overview

For the bacterial dataset, bioinformatic processing and data cleaning resulted in a total of 25,605 ESVs, of which 28% were assigned to the phylum Proteobacteria, 23% to Planctomycetota, and 8% to the SAR324 clade. A total number of 10,415 ESVs were obtained from the protistan dataset, with 35% assigned to the Alveolata clade, 23% grouped into the Rhizaria clade, and 18% assigned to the Stramenopiles clades, all three belonging to the SAR supergroup within the eukaryotic domain.

**Figure 2:**
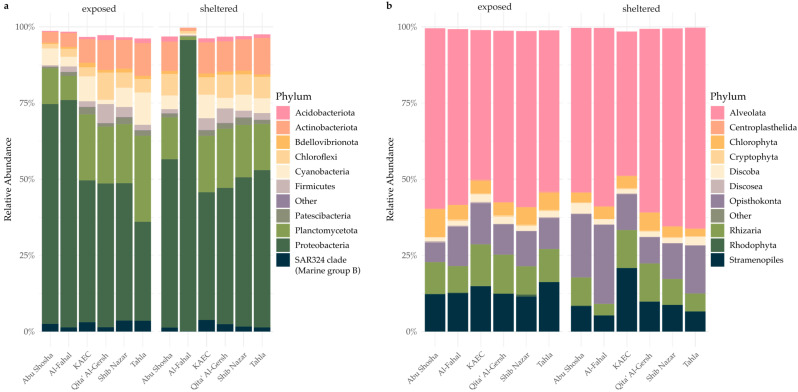
Relative abundance of microbial taxa at the phylum level for (**a**) bacteria and (**b**) protists across reefs and exposure sites. The stacked barplots show the mean relative abundance of phyla grouped by reef and exposure category (sheltered vs. exposed). Only the most abundant phyla are shown; low-abundance taxa (<1% across samples) are grouped under “Other”.

The taxonomic composition of reefs was homogenous within the bacterial communities, with the Abu Shosha and Al-Fahal reefs differing from the four remaining reefs in the relative abundance of the most dominant phyla and classes (Figure 2a). The protistan community compositions were even more homogenous (Figure 2b) and showed similar relative abundance patterns for the most abundant groups. Protistan communities overall showed a slight increase in the abundance of the Rhizaria phylum at exposed sites and a more pronounced shift within the Alveolata phylum for the Tahla and Abu Shosha reefs.

**Figure 3:**
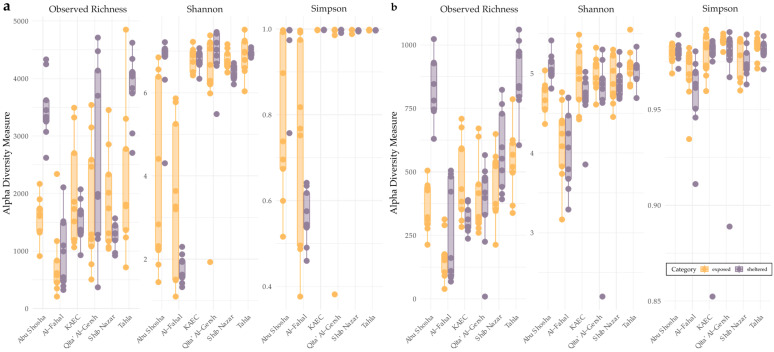
Alpha diversity of bacterial (**a**) and protist (**b**) communities across reefs and exposure sites. The boxplots show observed richness, Shannon diversity, and Simpson diversity indices for (**a**) bacteria and (**b**) protists at each reef, highlighted by sheltered (purple) and exposed (orange) sites. Each dot represents an individual sample.

Protistan and bacterial communities showed high richness and alpha diversity across Red Sea reefs, both at sheltered and exposed sites (Figure 3). Bacterial communities exhibited generally higher observed richness and alpha diversity compared to protistan communities. Notably, sheltered sites from the Al-Fahal reef showed reduced bacterial alpha diversity, while sheltered sites from the Tahla and Abu Shosha reefs displayed elevated protistan alpha diversity.

### 3.2. Impact of Reef and Exposure Site on Microbial Community Composition

Across the sampled coral reefs, alpha diversity analyses revealed pronounced within- and between-reef heterogeneity for both bacteria and protists. Kruskal–Wallis tests confirmed significant alpha diversity differences between reefs across both exposed (seaward) or sheltered (shoreward) categories. Pairwise Wilcoxon tests further revealed that observed richness and Shannon diversity differed significantly between multiple reef pairs across both microbial groups, particularly among Al-Fahal, Abu Shosha, and Tahla, regardless of whether samples were exposed or sheltered. For example, Al-Fahal (exposed) consistently diverged from other exposed reefs including Tahla, Shib Nazar, KAEC, and Qita’ Al-Gersh (adjusted *p* < 0.01). Similarly, Abu Shosha (both exposed and sheltered) showed repeated contrasts with other reefs in both microbial groups. These patterns were corroborated by beta diversity analyses, which detected significant compositional dissimilarities between reefs (between-reef PERMANOVA, bacteria: R^2^ = 0.27, F = 7.04, *p* = 0.001; protists: R^2^ = 0.15, F = 3.60, *p* = 0.001), confirming that reef identity was a major driver of microbial community structure.

Within-reef PERMANOVA also revealed significant differences in microbial community composition between exposed and sheltered samples across all six reefs (Table 2). The effect size varied by reef, with KAEC showing the strongest separation within both microbial groups while Qita’ Al-Gersh showed the weakest but still significant separation. Additionally, two-way PERMANOVA revealed significant reef × exposure interactions for both bacterial (R^2^ = 0.111, F = 3.29, *p* = 0.001) and protistan communities (R^2^ = 0.092, F = 2.35, *p* = 0.001), further solidifying that the influence of exposure (sheltered vs. exposed) varied across reefs. Residual variation remained high (65% and 75%, respectively), suggesting that additional unmeasured environmental or biotic factors contribute to community structure.

**Figure 4:**
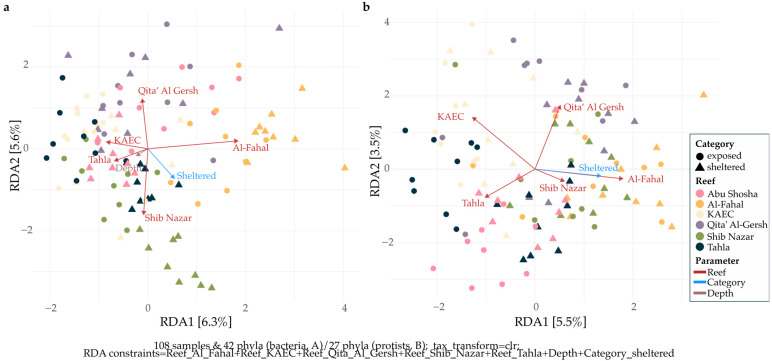
Redundancy analysis (RDA) of microbial community composition across Red Sea coral reefs and exposure categories. Panels show ordination results for bacterial (**a**) and protist (**b**) communities. Community data were CLR-transformed prior to ordination to account for the compositional nature of sequencing data. Points represent individual samples, colored by reef and shaped by category (sheltered vs. exposed). Axis labels indicate the proportion of variance explained by each ordination axis.

Redundancy analysis revealed that reef identity and exposure category significantly influenced bacterial and protistan community compositions (Figure 4). Additionally, for bacteria, depth was also a significant variable. Importantly, the analysis revealed similar patterns for both domains: communities inhabiting the reef Al-Fahal were most distinguishable, regardless of domain, and differed most from communities inhabiting the reef KAEC. Communities from the reef Qita’ Al Gersh fell in between the former two, with communities from the reefs Shib Nazar and Tahla being the most different from Qita’ Al Gersh communities. This indicates that individual reefs provide distinct ecological niches that shape communities across domains. However, while both RDAs were significant, the proportion of variance explained by the first two ordination axes was only 11.9% (bacteria) and 9% (protists), indicating that the majority of community variance is explained by other, non-measured abiotic and biotic factors.

Notably, while both alpha and beta diversity analyses converged in identifying reef identity and exposure category as key structuring factors, they also revealed nuanced differences. Beta diversity detected significant compositional shifts even in cases where Simpson diversity indicated no major changes in evenness, suggesting that subtle taxonomic turnover—not necessarily changes in dominant taxa—can drive community dissimilarity. Conversely, reefs showing strong differences in richness (e.g., via observed or Shannon metrics) also exhibited compositional dissimilarity, indicating a general alignment between taxonomic richness and broader community structure.

### 3.3. Microbial ESVs as Bioindicators

**Figure 5:**
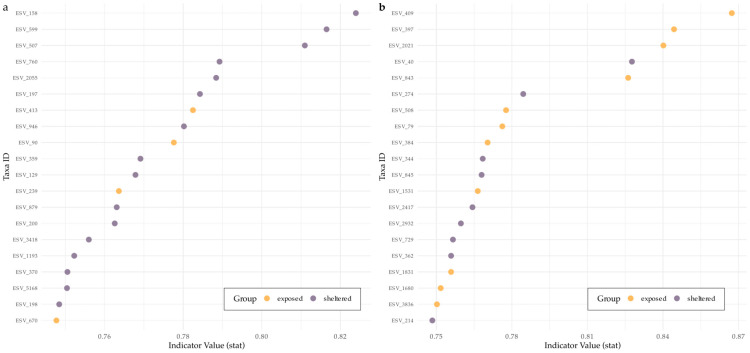
Top 20 indicator species for each exposure category identified by the IndVal method. (**a**) Bacterial ESVs and (**b**) protistan ESVs showing the highest indicator values (stat) based on association with either exposed or sheltered sites. Taxa are annotated at the phylum and species level and colored by the category with which they are significantly associated (*p* < 0.05). Indicator value (IndVal) reflects both the specificity and fidelity of each taxon to a particular exposure category.

Indicator species analysis revealed distinct sets of bacterial and protistan ESVs strongly associated with either sheltered or exposed sites. For both groups, we identified taxa with high indicator values (stat > 0.6), suggesting strong specificity and fidelity to the respective categories (Figure 5). For bacteria, ESV_158 assigned to the genus Methyloceanibacter showed the highest indicator values, followed closely by ESV_599 and ESV_507. However, no specific taxonomic group stood out as indicators (Figure 5a). For the protistan dataset, two ESVs assigned to the phylum Alveolata, two assigned to Rhizaria, and one to Opisthokonta exhibited the highest indicator values, with a noteworthy drop-off for the following ESVs (Figure 5b).

Random Forest models performed well for both datasets, with 500 trees built for each dataset and out-of-bag (OOB) errors of 12.04% for bacteria and 16.67% for protists, resulting in the accurate classifications of more than 80% of all samples. Of 54 exposed samples, 52 were classified correctly using the bacterial dataset, whereas only 45 were correctly classified for the protist dataset (Table 3a,b). For the 54 sheltered samples, 43 and 45 were classified correctly for the bacterial and protistan dataset, respectively (Table 3a,b).

**Figure 6:**
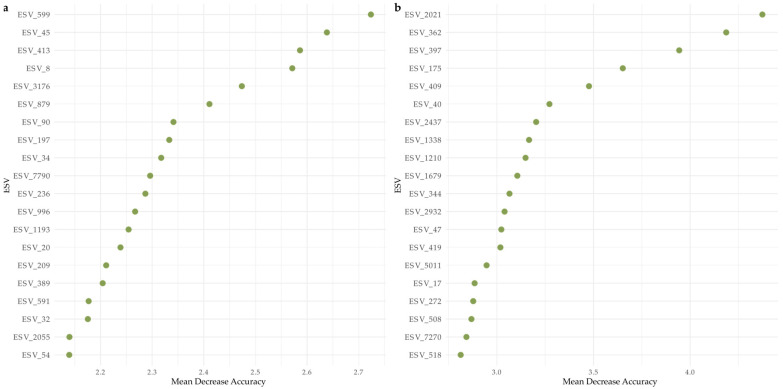
Variable importance based on Mean Decrease Accuracy (MDA) from Random Forest models classifying sheltered vs. exposed sites. Panel (**a**) shows the top bacterial ESVs, and panel (**b**) shows the top protistan ESVs ranked by their contribution to model accuracy. Higher MDA values indicate a greater loss in classification accuracy when the feature is permuted.

Model performance was higher when classifying exposed samples compared to sheltered samples for bacteria, whereas protists performed similarly for both exposure categories. Mean Decrease Accuracy (MDA) for bacteria (Figure 6a) and protists (Figure 6b) illustrate the importance of each ESV in classifying the samples, specifically showcasing how much the models’ accuracy would decrease with the removal of the respective ESVs from the datasets. For bacteria, specifically ESV_599 (Chloroflexi), ESV_45 (Cyanobacteria-Xenococcus sp.), ESV_413 (SAR324_clade-Marine Group B), and ESV_8 (Proteobacteria-Cohaesibacter sp.) were more influential in distinguishing exposed from sheltered sites. For protists, ESV_2021(Rhizaria-Miliolida), ESV_362 (Alveolata-Gregarinomorphea), ESV_175 (Alveolata-Colpodellidea), and ESV_409 (Alveolata-Vitrellaceae) had a significant contribution to model accuracy.

Overall, the indicator value approach and Random Forest classification shared several ESVs classified as “indicators”. For bacteria, five ESVs were identified by both approaches, whereas eight ESVs were identified by both approaches for the protistan dataset (Table 4a,b).

## 4. Discussion

Our study revealed clear multiscale patterns in bacterial and protist communities across coral reefs in the central Red Sea. By sampling both sheltered and exposed sites of six reefs, we demonstrated that microbial diversity and composition are structured not only by broad geographic differences between reefs, but also by finer-scale environmental variation within reefs. Specifically, sheltered (shoreward) and exposed (seaward) reef sites represent distinct microhabitats with exposed sites being characterized by higher water flow, greater light penetration, and reduced sediment accumulation, while sheltered sites exhibit calmer hydrodynamics, enhanced sedimentation, and greater temperature variability. These physicochemical contrasts provide distinct ecological microniches that strongly influence microbial community assembly.

### 4.1. Reef-Specific Bacterial and Protist Assemblages in the Red Sea

Beta diversity analyses showed significant compositional differences between reefs for both bacterial and protist communities, suggesting that each reef harbors a distinct microbial signature. These patterns likely reflect a combination of environmental filtering, stochastic colonization, and possibly host-associated community spillover from corals or macroalgae. The Red Sea is known for its pronounced local environmental gradients, including differences in wave energy, temperature microclimates, and benthic cover, which may create unique selective pressures at each exposure category.

This exposure specificity is consistent with findings from other marine ecosystems [51,52,53,54,55,56], where microbial communities often exhibit biogeographic structure despite the potential for wide dispersal [57].

For instance, studies in the Caribbean [58] and Indo-Pacific [59] have reported strong spatial turnover in reef microbial assemblages. Our results extend these observations to include protists, a group still understudied in reef microbiome research [4] but equally responsive to environmental heterogeneity.

### 4.2. Within-Reef Community Differences Reveal Microhabitat-Driven Divergence

Importantly, we also observed consistent differences between sheltered and exposed sites within the same reef. Alpha diversity was often significantly higher in one exposure category compared to the other, and beta diversity analyses showed that exposure drives community divergence even at this fine spatial scale. These results highlight the ecological relevance of reef microhabitats in structuring microbial communities. In sheltered sites, reduced water exchange and higher sedimentation can create environments enriched in organic matter that favor heterotrophic and anaerobic-associated taxa. In contrast, exposed sites with high water movement and light penetration select for photoautotrophic and mixotrophic taxa, alongside aerobic bacteria adapted to rapid organic matter turnover.

Sheltered and exposed reef sites differ in water quality, light penetration, sedimentation, and temperature variability owing to physical wave movement—all factors known to influence microbial composition [60,61]. Previous studies have documented shifts in microbial communities across depth and exposure gradients [10,62,63,64] including differences in, e.g., cyanobacterial abundance [65] and mixotrophic protists [66]. Our results reinforce the need to treat within-reef variation as an ecologically meaningful unit of study, particularly when designing microbial monitoring programs.

### 4.3. Cross-Domain Comparisons Reveal Overlapping but Non-Redundant Patterns

While bacterial and protist communities responded similarly to spatial structuring, they also exhibited domain-specific patterns. For example, certain reefs showed strong protist differentiation but weaker bacterial divergence, and vice versa. This highlights the complementary value of analyzing multiple microbial domains: while both groups are shaped by environmental conditions, their ecological niches and life strategies differ. Protists often function as grazers, phototrophs, or parasites [67], while bacteria dominate roles in decomposition, nutrient cycling, parasitism, and host symbiosis [63,68,69,70]. Combining both groups offers a more complete picture of microbial ecosystem dynamics covering multiple levels of the trophic cascade.

### 4.4. Indicator Taxa and Classification Models Support Community-Based Monitoring

Using indicator value analysis and Random Forest classification, we identified indicator taxa that reliably distinguish samples between reefs and exposure categories. These approaches are increasingly employed in microbial ecology to move beyond global patterns and toward actionable indicators. Many of the indicator taxa we identified belong to groups known to respond quickly to environmental change—including dinoflagellates [71] and ciliate protists [72,73].

Our results highlight habitat-specific microbial assemblages that may reflect environmental filtering or niche specialization. For example, bacterial taxa such as *Methyloceanibacter* and Chloroflexi clade members were associated with low-flow sheltered habitats, whereas members of the SAR324 clade were linked to exposed sites. Similarly, protistan indicators such as the alveolate classes Gregarinomorphea and rhizarian foraminifera (Miliolida) characterized sheltered microhabitats, while alveolate lineages like Vitrellaceae or ciliated *E. shenzhenense* were enriched in exposed sites. These patterns demonstrate how physicochemical conditions structure distinct microbial assemblages across reef microhabitats. Multiple indicator taxa were identified by both methods, making them strong bioindicator candidates. Additionally, the ability to classify samples into exposure categories using bioindicator taxa further highlights the differences in environmental characteristics between sheltered and exposed sites, such as water residency time and particulate matter suspension, which are likely to affect microbial communities [30].

While some indicator taxa were shared across microbial domains, others were specific to particular conditions, highlighting the potential of using multi-domain signatures for environmental assessment. Notably, the Random Forest models achieved higher classification accuracy compared to the indicator value approach, which corroborates with results from Frühe et al. [33], supporting the robustness of these taxa and methods as predictors of spatial structure. Such tools could be integrated into future reef monitoring frameworks to track ecosystem shifts or detect early signs of degradation.

The indicator taxa identified in our study reveal how sheltered and exposed reef microhabitats select for distinct microbial guilds with characteristic ecological functions. Among bacteria, *Methyloceanibacter* (Alphaproteobacteria) was strongly associated with sheltered sites. Members of this genus are known for methylotrophy, and their distribution patterns have been confirmed to be affected by carbon and nitrogen cycling and salinity, especially in coastal areas [74], traits that may be favored in high-flow, oxygenated environments where dissolved organic carbon is rapidly cycled. Similarly, SAR324 clade members, previously known as Marine Group B, are commonly detected in marine environments with a preference for deeper pelagic layers and broad metabolic versatility [75,76], and indicate exposed reef sites in this study. Chloroflexi (KD4-96), associated with heterotrophic degradation of organic matter in sediments [77], were enriched in sheltered sites with higher water residence time, less flushing, and higher sedimentation rates, indicating a potentially higher concentration of organic matter on the seafloor.

Several protistan lineages emerged as potential indicators of the contrasting microhabitats. Chromerids (e.g., Vitrellaceae) and foraminifera (Miliolida) were indicative of exposed reef sites. Chromerids are photosynthetic alveolates closely related to apicomplexans [78] and were originally isolated from corals [79]; their genomes and physiology endorse a phototrophic lifestyle compatible with high-light, oxygenated reef margins, where flow enhances nutrient exchange and reduces sediment resuspension (thus maintaining light availability). Likewise, miliolid foraminifera are classic inhabitants of high-light, carbonate-dominated shallow reefs, with their morphology designed for thriving under stable illumination and water movement [80]-conditions typical of exposed reef flats and crests. Their occurrence has long been used as a bioindicator of healthy reef systems [81,82]. Notably, another ESV assigned to the foraminiferal order of Miliolida in this study showed the highest indicator values for sheltered sites.

Taken together, these associations suggest that hydrodynamics and sedimentation not only structure microbial diversity but also filter for distinct metabolic strategies and morphological adaptions: rapid aerobic carbon cycling and phototrophy in exposed reefs versus particle-associated heterotrophy and mixotrophy in sheltered reefs. Such taxa may therefore serve as baseline bioindicators of microhabitat conditions, offering a functional lens through which to interpret microbial community change in coral reef ecosystems.

### 4.5. Implications and Future Directions

Together, our findings demonstrate that both bacteria and protists show structured, multiscale patterns of diversity in Red Sea coral reefs. These results underscore the value of including multiple microbial groups and sampling spatial gradients when studying reef microbiomes. The consistent within-reef differences suggest that small-scale heterogeneity—both ecologically and methodologically—matters and should be incorporated into sampling designs. More broadly, this work contributes to a growing effort of integrating microbial data retrieved from eDNA metabarcoding into coral reef monitoring and management. Future studies should investigate how these microbial patterns relate to environmental parameters (e.g., temperature, nutrients, benthic composition) and reef condition over time. The integration of eDNA, machine learning, and indicator-based frameworks holds promise for building predictive models that can inform reef conservation strategies in a rapidly changing ocean.

## Figures and Tables

**Table 1:** Target regions and primers used for eDNA amplification.

Primer	Gene	Target Taxa	References
16S27F534R	16S	Bacteria (BAC)	Muyzer et al. 1993, Weisburg et al. 1992 [34,35]
18SV9M	18S	Protists (PRO)	Stoeck et al. [36]

**Table 2:** PERMANOVA results for within-reef differences between exposed and sheltered samples for both microbial groups. * = 0.01 < *p* ≤ 0.05; ** = 0.001 < *p* ≤ 0.01; *** = *p* ≤ 0.001.

Reef	Bacteria		Protists	
	R^2^	*p*	R^2^	*p*
KAEC	0.41	0.001 ***	0.34	0.001 ***
Abu Shosha	0.35	0.001 ***	0.25	0.002 **
Tahla	0.28	0.002 **	0.11	0.014 *
Al-Fahal	0.17	0.008 **	0.13	0.003 **
Shib Nazar	0.13	0.001 ***	0.16	0.001 ***
Qita’ Al-Gersh	0.11	0.01 **	0.1	0.005 **

**Table 3:** (**a**) Confusion matrix of the Random Forest model for bacteria; (**b**) confusion matrix of the Random Forest model for protists.

(**a**)			
	Exposed	Sheltered	Class Error
Exposed	52	2	3.7%
Sheltered	11	43	20.4%
(**b**)			
	Exposed	Sheltered	Class Error
Exposed	45	9	16.67%
Sheltered	9	45	16.67%

**Table 4:** (**a**) Overview of bacterial ESVs identified by both the indicator value approach and Random Forest classification. Empty fields indicate incomplete taxonomic assignment; (**b**) overview of protistan ESVs found by both the indicator value approach and Random Forest classification. Empty fields indicate incomplete taxonomic assignment.

(**a**)							
ESV	Domain	Phylum	Class	Order	Family	Genus	
ESV_158	Bacteria	Proteobacteria	Alphaproteobacteria	Rhizobiales	Methyloligellaceae	*Methyloceanibacter*	
ESV_599	Bacteria	Chloroflexi	KD4-96	Uncult. bacterium			
ESV_2055	Bacteria	Actinobacteriota	Acidimicrobiia	Actinomarinales	Uncult. bacterium		
ESV_413	Bacteria	SAR324 clade(Marine Group B)	Uncult. bacterium				
ESV_879	Bacteria	Proteobacteria	Alphaproteobacteria	Rhodobacterales	Rhodobacteraceae	*Marinovum*	
(**b**)							
ESV	Domain	Phylum	Class	Order	Family	Genus	Species
ESV_409	Eukaryota	Alveolata	Colpodellidea	Vitrelladida	Vitrellaceae		
ESV_397	Eukaryota	Alveolata	Litostomatea	Haptoria_5	Pleurostomatida	*Epiphyllum*	*E. shenzhenense*
ESV_2021	Eukaryota	Rhizaria	Tubothalamea	Miliolida			
ESV_40	Eukaryota	Opisthokonta					
ESV_843	Eukaryota	Rhizaria					
ESV_1531	Eukaryota	Rhizaria	Globothalamea	Rotaliida			
ESV_2932	Eukaryota	Rhizaria	Tubothalamea	Miliolida	Moliolidae		
ESV_362	Eukaryota	Alveolata	Gregarinomorphea				

## Data Availability

All data analysis was performed in R_4.3.1(2023-06-16)–“Beagle Scouts” using RStudio_2023.06.1+524. All code and supporting files are publicly available and can be found on GitHub (https://github.com/lexscience/Molecular-Coral-Reef-Inventories). Sequence data was deposited in the Sequence Read Archive (SRA) of NCBI under project accession number PRJNA1000118.

## Supplementary Materials

The following supporting information can be downloaded at https://www.mdpi.com/article/10.3390/microorganisms13112549/s1: Table S1: Metadata of sampling locations in the central Red Sea.
